# First person – Evgeniya Karpova and Evgenii Komyshev

**DOI:** 10.1242/bio.056622

**Published:** 2020-10-08

## Abstract

First Person is a series of interviews with the first authors of a selection of papers published in Biology Open, helping early-career researchers promote themselves alongside their papers. Evgeniya Karpova and Evgenii Komyshev are co-first authors on ‘Quantifying *Drosophila* adults with the use of a smartphone’, published in BiO. Evgeniya is a postdoc in the lab of Nataly Gruntenko at the Research Center Institute of Cytology and Genetics, Siberian Branch of the Russian Academy of Sciences, investigating different aspects in insect neuro-hormonal stress response (on *Drosophila* model). Evgenii is a PhD student in the lab of Dmitry Afonnikov at the Research Center Institute of Cytology and Genetics, Siberian Branch of the Russian Academy of Sciences, investigating information technologies in genetics.


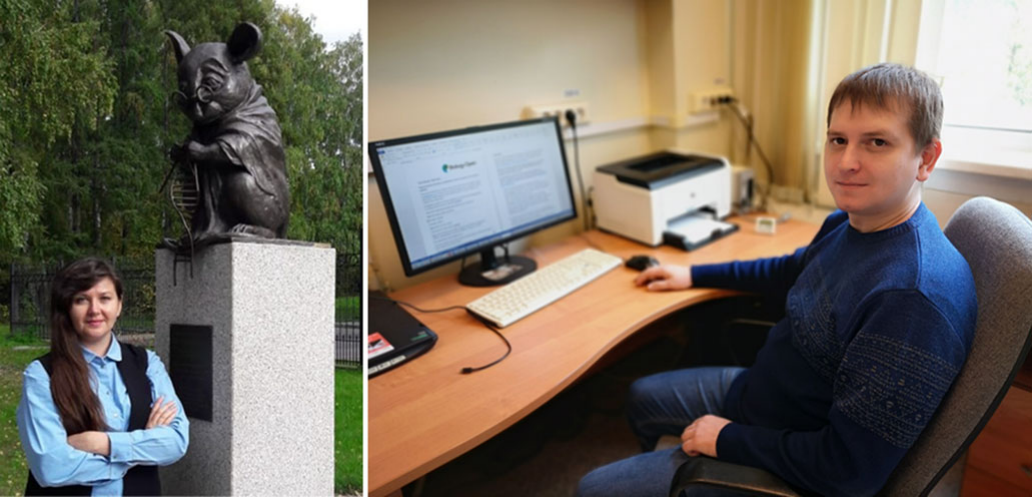


**Evgeniya Karpova and Evgenii Komyshev**

**What is your scientific background and the general focus of your lab?**

**E.K.K.:** I am a postdoctoral researcher in the laboratory of stress genetics led by Nataly Gruntenko at the Research Center Institute of Cytology and Genetics, Siberian Branch of the Russian Academy of Sciences, investigating genetic and physiological basis of stress response to *Drosophila* as a model.

**E.G.K.:** I am a PhD student in the sector of Bioinformatics and Information Technologies in Genetics led by Dmitry Afonnikov at the same research center.

**E.K.K. & E.G.K.:** We work in different areas, and the combination of experimental and technical expertise allowed us to develop a method for automation of imago quantifying and fecundity assessment in *Drosophila* with the use of mobile devices running the Android operating system.

**How would you explain the main findings of your paper to non-scientific family and friends?**

**E.K.K.:** The synthetic theory of evolution understands fitness as reproductive success, i.e. the ability of an individual to produce offspring and thus transfer its genes. This is why fecundity is the most frequently used parameter for the estimation of fitness in insects. Although offspring calculation is conceptually simple, in practice it is a laborious task. We optimized, tested, and introduced a new method of automated count of *Drosophila* adults using the SeedCounter mobile application. The application is available on all mobile devices based on the Android system, and it does not require any additional equipment. It makes accurate calculations and can seriously save time for anyone who faces the unenviable task of counting fecundity in flies.

**E.G.K.:** This application allows users to quickly and accurately calculate the number of offspring in a wide range of values. The estimates of flies fecundity obtained with the use of the mobile app were compared with the results of manual counts of the same samples. The data show a good correspondence of the number of flies obtained manually and with the help of the mobile app, which indicates its high accuracy and efficiency.

**What are the potential implications of these results for your field of research?**

**E.K.K.:** The traditional manual method of counting the progeny takes a long time and limits the opportunity of making large-scale experiments. A development of computer methods that would allow us to automatically make a quantitative estimate of *Drosophila melanogaster* fecundity is an urgent task. With the use of the mobile application SeedCounter that analyzes images of objects placed on a standard sheet of paper, we would be able to study *D. melanogaster* fecundity in large samples. The method would also be useful for quantifying numbers of adult flies in longevity or viability experiments.

**E.G.K.:** Surprisingly, the SeedCounter application, which was originally developed for counting and measuring wheat grains, performed well in the task of counting *D. melanogaster* flies. The changes made to the application to process *D. melanogaster* images were quite small. This gives us reason to believe that our application can be used to count other biological objects of the similar size.

**What has surprised you the most while conducting your research?**

**E.K.K.:** I was amazed at how much modern technique can facilitate the work of an ordinary biologist. You must always try to find opportunities to use new techniques to improve the efficiency of scientific work and never stop there.

**E.G.K.:** I was surprised that in the digital images we captured, you can estimate the body shape of *D. melanogaster*, which in turn could be used to determine their gender in the future.

“I was amazed at how much modern technique can facilitate the work of an ordinary biologist.”

**What, in your opinion, are some of the greatest achievements in your field and how has this influenced your research?**

**E.K.K. & E.G.K.:** Recently the methods using mobile devices for analyzing the images of biological objects have developed rapidly. Modern mobile devices (smartphones and internet tablets) have high resolution digital cameras and multi core processors with enough processing power for image processing and analysis. These functions allow users to take and process images where it is necessary and make a rapid and accurate count. We have taken this as our inspiration to suggest a method that helps us to measure fecundity by adult progeny emergence in *Drosophila* with the use of mobile devices.

**Figure:**
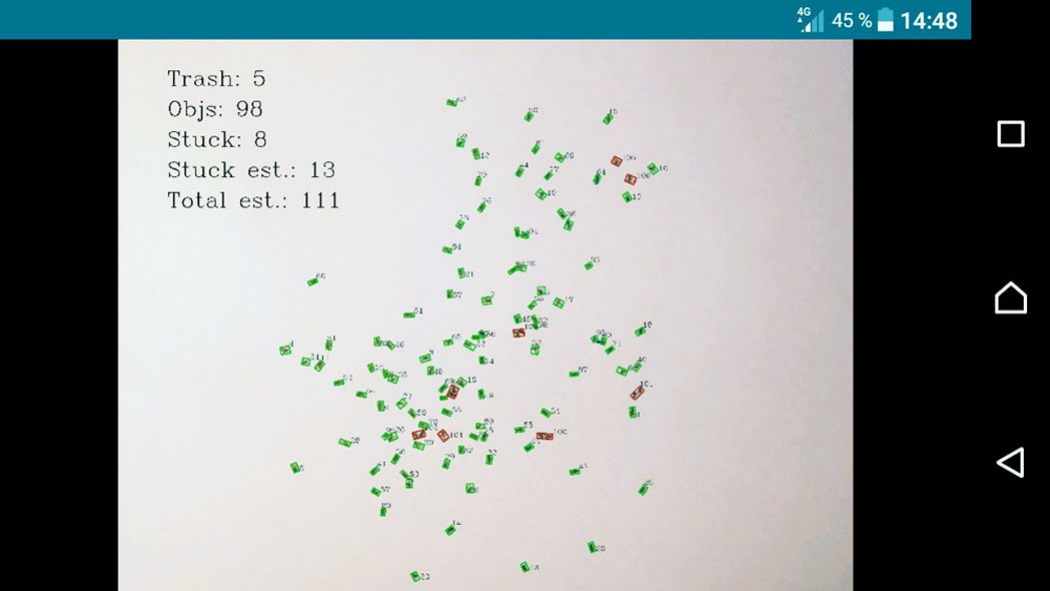
**Evgenia Karpova calculates the fertility of *D. melanogaster* using the SeedCounter program.**

**What changes do you think could improve the professional lives of early-career scientists?**

**E.K.K.:** In my opinion, the main thing for a young scientist is to choose an interesting topic that will inspire him or her to research and benefit all of humanity. However, early career researchers lack experience. A friendly team and effective communication with colleagues can help young researchers learn a lot and move in the right direction.

**E.G.K.:** In my opinion, the professional lives of early-career scientists could improve the expanding introduction of new technologies and the use of modern tools, which are currently appearing more and more. Modern technologies allow you to measure and evaluate what was previously impossible, or too labor-intensive. These innovations can significantly facilitate routine operations, as well as improve the accuracy, objectivity and efficiency of modern experiments.

**What's next for you?**

**E.K.K.:** I hope the new method will increase the quality and speed of my research in the field of adaptation to adverse environmental conditions. In particular, the detection of long-term effects of regular stress exposure in the *D. melanogaster* model. In the future, these studies can help to find the way to improve stress resistance in different biological objects including humans, which is very important in our time.

**E.G.K.:** We plan to improve our app: expand its functionality and availability, and improve its usability. We hope that as many researchers as possible will discover SeedCounter as a useful tool in their work.
